# ROS Production and Distribution: A New Paradigm to Explain the Differential Effects of X-ray and Carbon Ion Irradiation on Cancer Stem Cell Migration and Invasion

**DOI:** 10.3390/cancers11040468

**Published:** 2019-04-03

**Authors:** Anne-Sophie Wozny, Guillaume Vares, Gersende Alphonse, Alexandra Lauret, Caterina Monini, Nicolas Magné, Charlotte Cuerq, Akira Fujimori, Jean-Claude Monboisse, Michael Beuve, Tetsuo Nakajima, Claire Rodriguez-Lafrasse

**Affiliations:** 1 Laboratoire de Radiobiologie Cellulaire et Moléculaire, Faculté de Médecine Lyon-Sud, Univ Lyon, Université Lyon 1, UMR CNRS5822/IN2P3, IPNL, PRISME, 69921 Oullins CEDEX, France; anne-sophie.wozny@univ-lyon1.fr (A.-S.W.); gersende.alphonse@univ-lyon1.fr (G.A.); alexandra.lauret@univ-lyon1.fr (A.L.); nicolas.magne@icloire.fr (N.M.); 2 Centre Hospitalier Lyon-Sud, Service de Biochimie et Biologie Moléculaire, Hospices Civils de Lyon, 69495 Pierre-Bénite, France; charlotte.cuerq@chu-lyon.fr; 3 Advanced Medical Instrumentation Unit, Okinawa Institute of Science and Technology Graduate University, Okinawa 904-0495, Japan; guillaume.vares@oist.jp; 4 Department of Radiation Effects Research, National Institute of Radiological Sciences, National Institute for Quantum and Radiological Science and Technology, Inage-ku, Chiba 263-8555, Japan; nakajima.tetsuo@qst.go.jp; 5 Univ Lyon, Université Lyon 1, UMR CNRS5822 /IN2P3, IPNL, PRISME, PHABIO, 69322 Villeurbanne, France; monini@ipnl.in2p3.fr (C.M.); m.beuve@ipnl.in2p3.fr (M.B.); 6 Département de Radiothérapie, Institut de Cancérologie de la Loire Lucien Neuwirth, 42270 St Priest en Jarez, France; 7 Department of Basic Medical Sciences for Radiation Damages, National Institute of Radiological Sciences, Inage-ku, 263-8555 Chiba, Japan; fujimori.akira@qst.go.jp; 8 Université de Reims Champagne-Ardenne, CNRS UMR 7369, CHU de Reims, 51100 Reims, France; jean-claude.monboisse@univ-reims.fr

**Keywords:** X-ray irradiation, carbon ion irradiation, reactive oxygen species, cancer stem cells, hypoxia, metastases, invasion/migration

## Abstract

Although conventional radiotherapy promotes the migration/invasion of cancer stem cells (CSCs) under normoxia, carbon ion (C-ion) irradiation actually decreases these processes. Unraveling the mechanisms of this discrepancy, particularly under the hypoxic conditions that pertain in niches where CSCs are preferentially localized, would provide a better understanding of the origins of metastases. Invasion/migration, proteins involved in epithelial-to-mesenchymal transition (EMT), and expression of MMP-2 and HIF-1α were quantified in the CSC subpopulations of two head-and-neck squamous cell carcinoma (HNSCC) cell lines irradiated with X-rays or C-ions. X-rays triggered HNSCC-CSC migration/invasion under normoxia, however this effect was significantly attenuated under hypoxia. C-ions induced fewer of these processes in both oxygenation conditions. The differential response to C-ions was associated with a lack of HIF-1α stabilization, MMP-2 expression, or activation of kinases of the main EMT signaling pathways. Furthermore, we demonstrated a major role of reactive oxygen species (ROS) in the triggering of invasion/migration in response to X-rays. Monte-Carlo simulations demonstrated that HO^●^ radicals are quantitatively higher after C-ions than after X-rays, however they are very differently distributed within cells. We postulate that the uniform distribution of ROS after X-rays induces the mechanisms leading to invasion/migration, which ROS concentrated in C-ion tracks are unable to trigger.

## 1. Introduction

Cancer stem cells (CSCs), a subpopulation within the bulk tumor, are intimately involved in radio- and chemo-resistance, self-renewal and clonal growth via the generation of a non-stem-cell populations [1]. A growing body of evidence has demonstrated that CSCs may have the phenotypic hallmarks of mesenchymal cells [2] and that there is a relationship between the presence of CSCs and metastasis [3]. Further, CSCs are located in hypoxic niches which enable them to maintain stemness and also promote epithelial-to-mesenchymal transition (EMT) [4].

Hadron therapy with carbon ions (C-ions) is known to have physical and biological advantages over conventional radiotherapy for treating radioresistant tumors [5]. The precise energy deposition (Bragg peak) enables deep-seated tumors to be targeted while sparing the surrounding tissues [6]. Furthermore, in contrast to X-ray irradiation where ionization is spread uniformly inside cells, C-ions produce a very high ionization density within individual tracks. This high dose induces complex and unrepairable DNA damage leading to cell death [7]. Several studies have reported that C-ions kill CSCs better than conventional radiotherapy [8,9,10]. Moreover, most of the time, although X-rays promote migration and invasiveness under normoxia [11], C-ions produce a significant benefit by decreasing the migration and invasiveness of cancer cells in vitro [11,12] and in vivo [13,14]. Conversely, a few studies report anti-invasive capacities in response to X-rays, whereas C-ions can be pro-invasive [15]. Another advantage of C-ion irradiation is that, unlike X-ray irradiation, it induces biological effects independent of the tumor oxygen concentration, which results in lower oxygen enhancement ratios [16].

Hypoxia-inducible factor-1α (HIF-1α) is the main transcription factor involved in the cellular response to hypoxia [17] and is associated with metastases, resistance to therapy and poor survival [18]. Its role in the activation of the EMT has already been described [19]: e.g., Gu et al. have demonstrated that X-ray irradiation promotes EMT in non-small-cell lung carcinoma through the activation of the HIF-1α pathway. However, no expression of HIF-1α occurs after C-ion exposure under normoxic conditions [9,20].

Upstream of HIF-1α, three main signaling pathways (STAT3, Akt/mTOR and MAPK) involved in both migration/invasion and EMT are modulated by radiation [21,22]. However, although less migration/invasion has been reported with C-ion irradiation compared with X-ray irradiation under normoxic conditions [12], few data are available regarding the molecular response to X-rays under hypoxic conditions [23,24] and none regarding the response to C-ions in hypoxic conditions.

Therefore, this work aimed to characterize the mechanisms leading to the inhibition of migration/invasion in head-and-neck squamous cell carcinoma (HNSCC)-CSCs after C-ion irradiation, particularly under hypoxic conditions, with a specific focus on the role of reactive oxygen species (ROS), HIF-1α and the upstream signaling pathways.

## 2. Results

### 2.1. Impact of Oxygenation Conditions on Cell Motility, Migration and Invasiveness of CSCs Versus Non-CSCs

**Figure 1:**
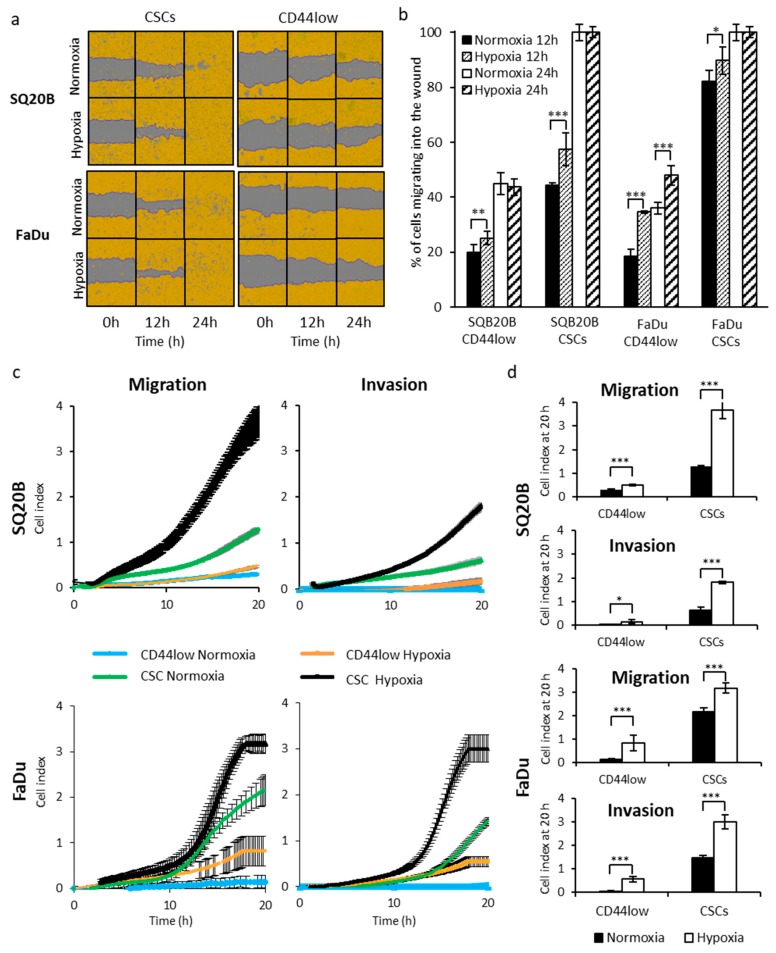
Effect of hypoxia on the motility, cell migration and invasiveness of head-and-neck squamous cell carcinoma (HNSCC)-cancer stem cells (CSCs) and non-CSC subpopulations. (**a**) Representative images of wound-healing assay obtained using IncuCyte at 0, 12 and 24 h after cell scraping in normoxic or acute hypoxic conditions (*n* = 2, triplicates). (**b**) Quantification of the wound rate recovery in normoxia and acute hypoxia, 12 h after cell scraping. Significance was assessed for hypoxia compared with the corresponding normoxic condition (* *p* < 0.05, ** *p* < 0.01, *** *p* < 0.005, Student’s *t* test). (**c**) Real-time analyses of cell migration/invasion under normoxia or hypoxia followed over 20 h with xCELLigence (mean ± SD of 4 wells), *n* = 3. (**d**) Migration and invasion cell index obtained with xCELLigence at 20 h in normoxia and hypoxia. Normoxic and correspondent hypoxic cell index were compared using Student’s *t* test.

The motility of SQ20B-CSCs and FaDu-CSCs and their corresponding non-CSCs (SQ20B^CD44low^ and FaDu^CD44low^) was quantified under normoxic and hypoxic conditions using a wound-healing assay (IncuCyte; Essen Bioscience, Hertfordshire, UK) (Figure 1a).

The quantification of wound recovery revealed that CSCs had a much better ability to close the wound than non-CSCs (Figure 1b). Under normoxia, both CSC subpopulations were able to totally close wounds by 24 h after they were made, whereas only 45 ± 4% of SQ20B^CD44low^ and 36 ± 2% of FaDu^CD44low^ cells migrated into the wound area. Hypoxia significantly increased the proportion of cells from all four populations that entered the wound 12 h after it was created (Student’s *t* test *** *p* < 0.005).

Cell migration across a 2D substrate and invasion through a 3D basement-membrane-extract biomatrix in response to a serum gradient was then visualized in real-time using xCELLigence (Roche, Mannheim, Germany) (Figure 1c) and quantified 20 h after cell seeding (Figure 1d). Under normoxia, invasion of the four cell subpopulations through the matrix was lower than their migration. Under hypoxia, invasion and migration were greater than those under normoxia for all four subpopulations (e.g., the mean ± SD cell index signals for invasion were 0.60 ± 0.13 for SQ20B-CSCs under normoxia versus 1.82 ± 0.05 under hypoxia and 1.44 ± 0.11 for FaDu-CSCs versus 3.00 ± 0.3 under hypoxia), and this difference was greater for CSCs than for non-CSCs.

These first results demonstrate that CSCs are the subpopulation with the greatest ability to migrate and invade, particularly in hypoxic conditions.

### 2.2. Influence of Oxygenation Conditions on the Expression of EMT- and Stemness-Related Markers in CSCs Versus Non-CSCs

**Figure 2:**
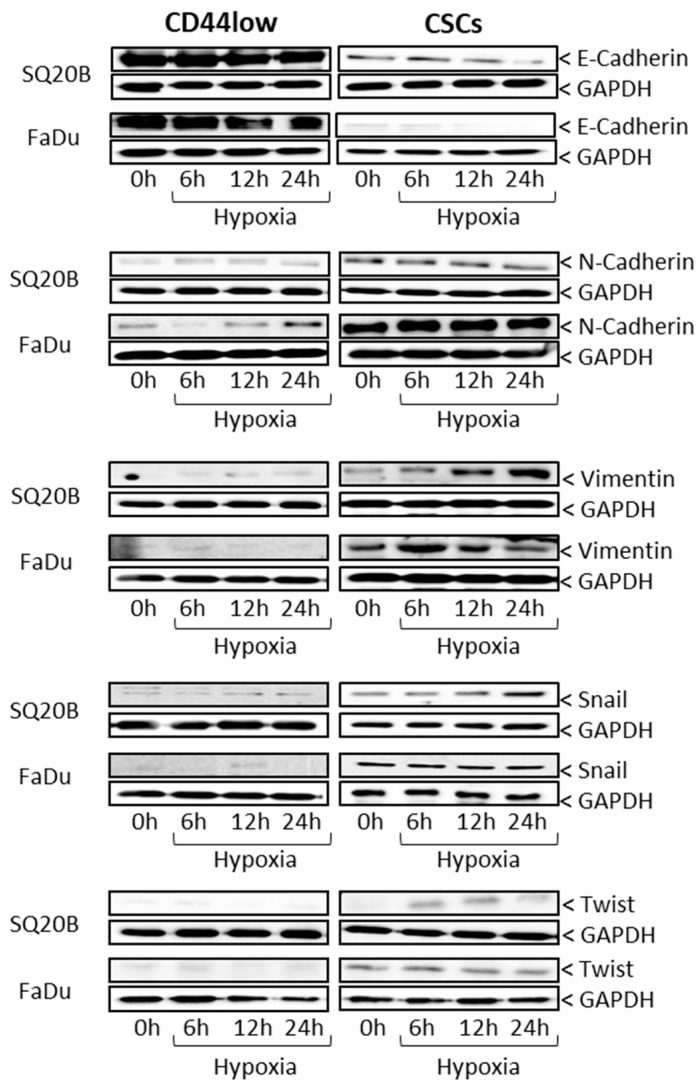
Impact of acute hypoxia on the epithelial-to-mesenchymal transition (EMT) phenotypes of CSCs and non-CSCs. Representative Western blots of epithelial markers (E-cadherin), mesenchymal markers (N-cadherin, vimentin) and stemness markers (Snail, Twist) in the four HNSCC subpopulations under normoxia (0 h) and after 6, 12 and 24 h of acute hypoxia (*n* = 3, Supplementary Figure S1).

The expression of proteins reflecting the epithelial (E-cadherin) and mesenchymal (N-cadherin, vimentin) phenotypes as well as stemness (Snail, Twist) were studied using Western blotting (Figure 2) under normoxia (0 h) or after 6, 12 and 24 h of acute hypoxia.

Under normoxia and hypoxia, both CSC subpopulations exhibited a mesenchymal phenotype (i.e., elevated expression of N-cadherin and vimentin and low expression of E-cadherin) compared with non-CSCs, which displayed an epithelial phenotype (lower levels of N-cadherin and vimentin and higher levels of E-cadherin). Although no significant expression of the stemness markers (Snail and Twist) was found for non-CSCs in either oxygenation condition, an increase in the expression of these proteins was observed for CSCs under hypoxia. These data confirm the relationship between the mesenchymal phenotype of CSCs and their capacity for invasion/migration.

### 2.3. Consequences of X-ray or C-ion Irradiation on Cell Migration and Invasion under Normoxia or Chronic Hypoxia

**Figure 3:**
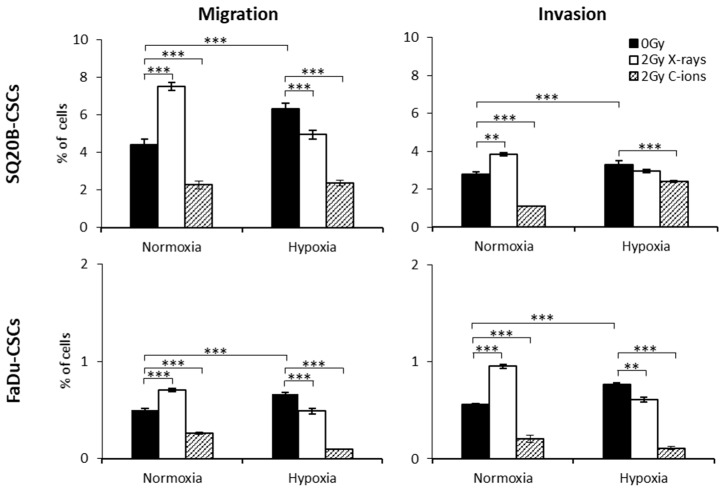
Effect of X-ray and C-ion irradiation on migration/invasion processes under normoxia and hypoxia. Three independent experiments using Boyden chambers were performed in triplicate (mean ± SD of the percentage of SQ20B-CSCs and FaDu-CSCs that migrated into the chambers) after 2 Gy X-ray or C-ion irradiation and compared with those using sham-irradiated cells (0 Gy) under normoxia or chronic hypoxia (* *p* < 0.05, ** *p* < 0.01, *** *p* < 0.005, Student’s *t* test).

As IncuCyte was not available at the National Institute of Radiological Sciences (C-ion beam, NIRS, Chiba, Japan), Boyden chamber assays were used to investigate the effect of C-ion and X-ray irradiation on CSC subpopulations (Figure 3). In normoxia, 2 Gy X-ray irradiation significantly enhanced the abilities of CSCs to migrate and invade. Under hypoxia, the basal level of migration/invasion was significantly higher than that under normoxia. Exposure of CSCs to X-rays under hypoxia decreased these processes compared with hypoxia without irradiation. In response to 2 Gy C-ion irradiation, a significant decrease in the migration/invasion of SQ20B-CSCs and FaDu-CSCs was observed under both conditions of oxygenation. These results indicate that C-ion irradiation decreases the migration/invasion of CSCs to a greater extent than a physically equivalent dose of photons, regardless of the degree of oxygenation.

### 2.4. MMP-2 and MMP-9 Levels in Response to X-Ray and C-Ion Irradiation

**Figure 4:**
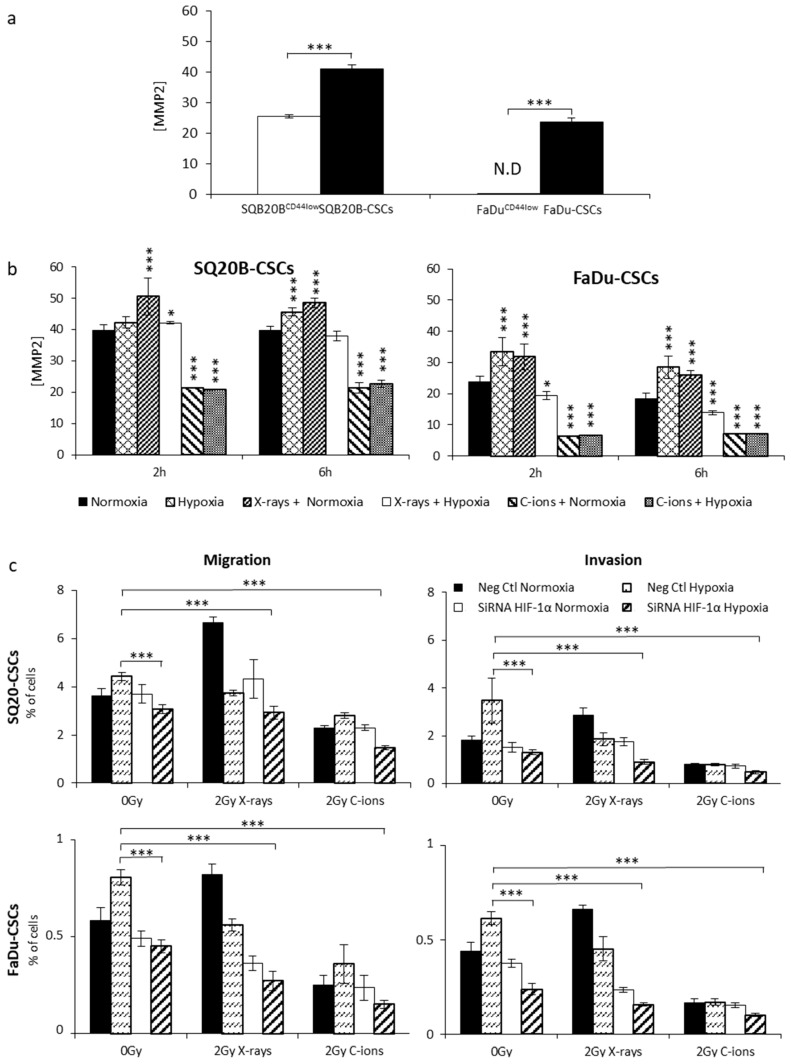
Effect of X-ray and C-ion irradiation on migration/invasion processes under normoxia and chronic hypoxia. (**a**) MMP-2 concentrations in SQ20B and FaDu subpopulations normalized to baseline protein levels; *n* = 3. (**b**) MMP-2 concentrations in SQ20B-CSCs and FaDu-CSCs measured 2 and 6 h after both types of irradiation (10 Gy) under normoxia and chronic hypoxia or under hypoxia alone. Each condition was performed in triplicate and compared to normoxia using Student’s *t* test. (**c**) Boyden chamber assays were performed in triplicate for SQ20B-CSCs and FaDu-CSCs in response to 2 Gy X-ray (*n* = 3) or C-ion (*n* = 2) irradiation with or without HIF-1α silencing. The siRNA conditions were compared with control cells under normoxia (* *p* < 0.05, ** *p* < 0.01, *** *p* < 0.005, Student’s *t* test).

To confirm the differential effect on migration and invasion processes observed after X-ray and C-ion irradiation, the concentrations of MMP-2 and MMP-9, two major metalloproteases involved in the degradation of the extracellular matrix, were quantified in the different experimental conditions. MMP-9 was undetectable with the Quantikine Total MMP-9 Immunoassay kit in all the tested conditions. Under normoxia, MMP-2 levels (normalized to total protein; Figure 4a) were significantly higher in CSCs (mean ± SD: 41.2 ± 1.3 for SQ20B and 23.7 ± 1.3 for FaDu) than in non-CSCs (25.5 ± 0.5 and undetectable, respectively).

A significant increase in the MMP-2 concentrations was measured for both CSC populations in response to hypoxia without irradiation and under normoxia with 10 Gy X-ray irradiation (Figure 4b). As expected, a significant decrease of MMP-2 (** *p* < 0.001) was quantified after 10 Gy C-ion irradiation in both oxygenation conditions, which is consistent with the decrease in invasion/migration decrease described above.

### 2.5. Involvement of HIF-1α in the Migration/Invasion Abilities of Irradiated CSCs

We previously showed that HIF-1α is expressed in response to photons in normoxic and hypoxic conditions, whereas in response to C-ions, only the hypoxic condition led to HIF-1α stabilization. To determine whether HIF-1α expression could be responsible for the differential pattern of responses to X-ray and C-ion irradiation, we performed expression silencing experiments (two siRNAs and a negative control) in SQ20B-CSCs and FaDu-CSCs (Figure 4c) before performing Boyden chamber assays. In normoxic non-irradiated cells, the silencing of HIF-1α did not significantly modify the proportion of migrating and invading cells. In hypoxic non irradiated cells, inhibiting HIF-1α expression significantly decreased both migration and invasion. Under normoxia, the combination of 2 Gy X-rays with HIF-1α silencing decreased the migration/invasion. These processes were also reduced under hypoxia combined or not with irradiation. After C-ion irradiation in normoxia, the inhibition of HIF-1α did not significantly modify the proportion of migration/invasion compared with negative control cells. Interestingly, under hypoxia, the inhibition of HIF-1α associated with C-ion exposure led to a significant reduction in cell migration and invasion compared with the non-irradiated negative control, suggesting that the decrease in these processes was essentially linked to the effect of hypoxia.

Overall, these results demonstrate a major role of HIF-1α in the invasion/migration processes, particularly in response to X-ray under hypoxic conditions.

### 2.6. Regulators of the Migration/Invasion Processes Following Photon or C-ion Irradiation

**Figure 5:**
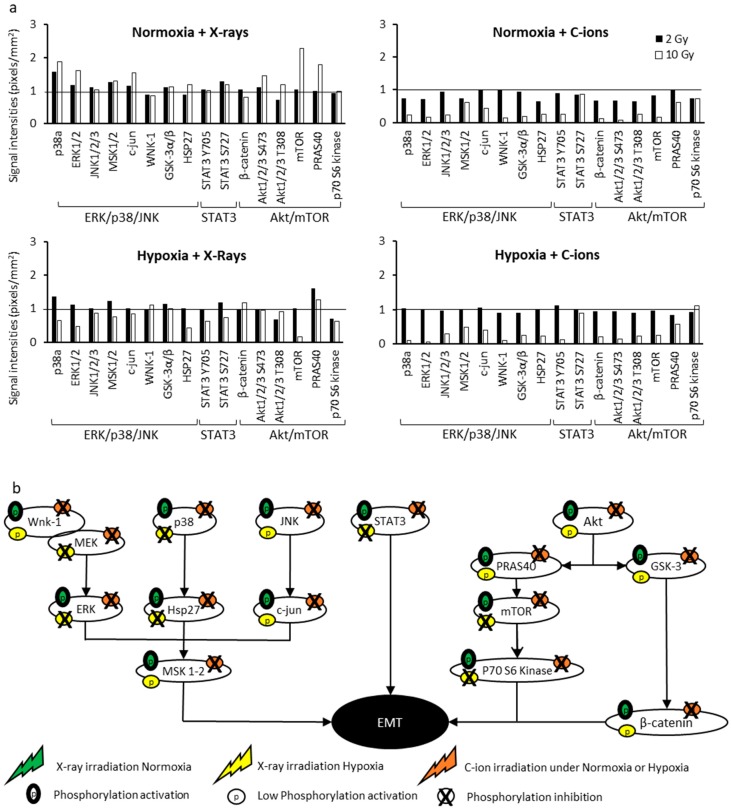
Signaling pathways involved in the migration/invasion processes. (**a**) The phosphorylation levels of the proteins involved in the MEK/p38/JNK, STAT3 and Akt/mTOR pathways were determined for SQ20B-CSCs in response to 2 or 10 Gy X-rays and C-ion irradiation ± chronic hypoxia, and normalized to basal conditions using the Proteome Profiler Human Phospho-Kinase Array Kit. A signal > 1 corresponded to an activation of phosphorylation, whereas a signal < 1 was associated with an inactivation of the kinases (*n* ≥ 2 in duplicate). (**b**) Schematic representation of the MEK/p38/JNK, STAT3 and Akt/mTOR pathways involved in migration/invasion under normoxia and hypoxia as a function of the type of irradiation.

We next focused on the regulation of three main signaling pathways involved in the migration/invasion processes, namely ERK/p38/JNK, Akt/mTOR and STAT3. The phosphorylation levels of 16 kinases in SQ20B-CSCs 6 h after irradiation with 2 or 10 Gy of X-ray or C-ions were quantified using the Proteome Profiler Human Phospho-Kinase Array Kit (R&D Systems) and normalized to their basal levels in normoxia (Figure 5a). Experiments were also performed in SQ20B^CD44low^ at 10 Gy (Supplementary Figure S2). The raw results for SQ20B-CSCs at 2 and 10 Gy are reported in Supplementary Tables S1 and S2, respectively, and those for SQ20B^CD44low^ at 10 Gy in Supplementary Table S3. In response to 2 and 10 Gy X-rays under normoxia, 12 and 13, respectively, of the 16 kinases were activated in SQ20B-CSCs, as evidenced by ratios > 1. Only 9 of the 16 kinases were activated in the SQ20B^CD44low^ cells irradiated with 10 Gy (Supplementary Figure S2). In SQ20B-CSCs in response to X-rays under hypoxia, the phosphorylation ratios were > 1 for 11 kinases at 2 Gy and only 4 kinases at 10 Gy. None of the three pathways were activated in SQ20B^CD44 low^ cells, consistent with their lower ability to migrate and invade compared with CSCs. After 2 and 10 Gy C-ion irradiation under both oxygenation conditions, a major decrease in the phosphorylation signals, as shown by a ratio lower than < 1, was present for the majority of the kinases. Figure 5b summarizes the phosphorylation status of the major kinases involved in the three signaling pathways of invasion/migration in the different conditions of irradiation. These data show the activation by phosphorylation of several different kinases involved in the invasion/migration processes after X-rays, but importantly, the absence of kinase activation in response to C-ion irradiation.

### 2.7. ROS-Dependence of Migration and Invasion Processes

**Figure 6:**
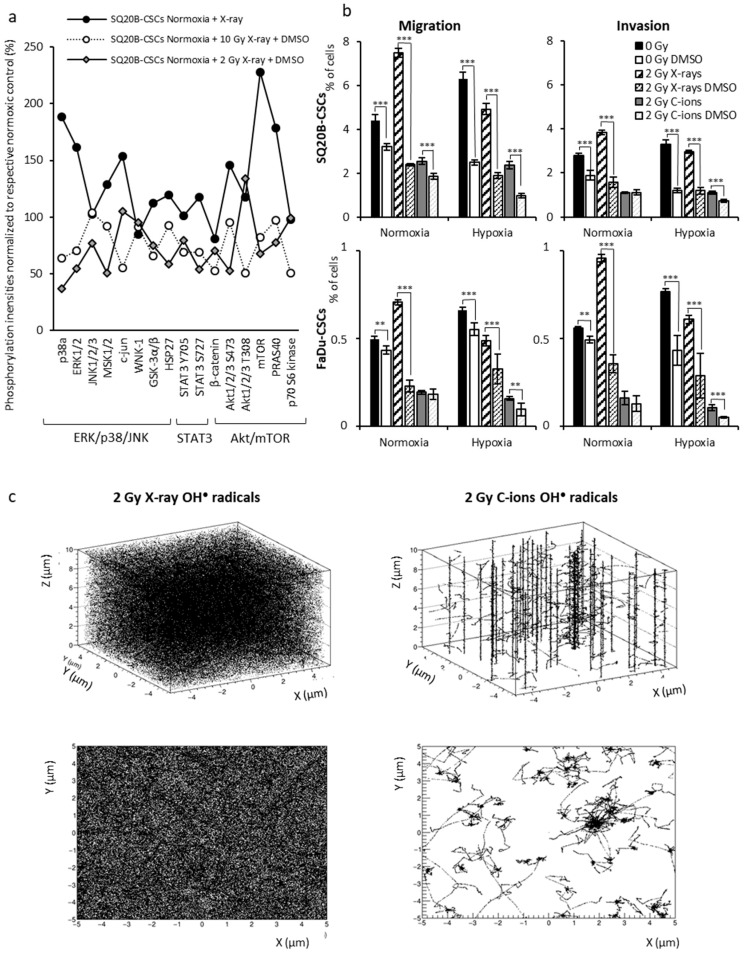
Impact of ROS production on CSC migration and invasion. (**a**) Phosphorylation signals obtained using the Proteome Profiler Human Phospho-Kinase Array Kit were normalized to those in normoxia for SQ20B-CSCs with or without treatment with 1% DMSO after 2 Gy or 10 Gy X-ray irradiation (*n* ≥ 2 in duplicate). (**b**) Boyden chamber assays were performed in SQ20B-CSCs and FaDu-CSCs after 1% DMSO treatment ± 2 Gy X-ray or C-ion irradiation under normoxia or chronic hypoxia. (* *p* < 0.05, ** *p* < 0.01, *** *p* < 0.005, Student’s *t* test) (*n* = 3). (**c**) HO^●^ radical distribution in response to X-rays and to a mixed radiation field reproducing the NIRS irradiation in C-ions Spread-Out Bragg Peak (SOBP) (dose-averaged Linear Energy Transfer (LET) ~13 keV/µm).

ROS could be suitable candidates to explain the relationship between X-ray irradiation and the activation of signaling pathways. When ROS production was inhibited with DMSO (a preferential scavenger of hydroxyl radicals) after 2 Gy and 10 Gy X-ray irradiation and particularly under normoxic conditions, the activation of the signaling pathways studied above decreased to 38% and 58%, respectively, of the control level in SQ20B-CSCs, confirming that ROS play a key role in invasion/migration mechanisms through MAPK, STAT3 and Akt/mTOR pathway signaling (Figure 6a; Supplementary Tables S4 and S5). Experiments were also performed for SQ20B^CD44low^ cells and showed a lesser impact of ROS scavenging on the phosphorylation of kinases than in SQ20B-CSCs (Supplementary Table S6).

As shown in Figure 6b, treatment with DMSO without irradiation significantly reduced the proportion of cells migrating and invading the matrix in normoxia, however this decrease was exacerbated under hypoxia. The scavenging of ROS significantly reduced migration/invasion after X-ray irradiation in both conditions of oxygenation. As hypothesized, ROS scavenging did not significantly influence the response of cells to C-ion irradiation. These results demonstrate that ROS play an essential role in the signaling and triggering of migration/invasion processes in response to X-rays, the effect being greater under normoxia, but not in response to C-ion irradiation.

### 2.8. ROS Distribution in Response to X-ray and C-ion Irradiation

In light of the above results, we hypothesized that it is the different spatial distribution of ROS after the two types of irradiation rather than their relative concentration that explains the discrepancies in terms of migration/invasion and signaling described above. To demonstrate this, we performed Monte Carlo simulations of hydroxyl (OH^●^) species generation in water because OH^●^ is the most reactive ROS. We compared the distributions obtained in response to X-rays and to a mixed radiation field with an averaged-dose LET corresponding to the experimental mean value of 13 keV/µm (NIRS irradiation in C-ions SOBP). Because 10 µm represents the order of magnitude of the cell dimensions considered, we set the irradiated volume as a 10 µm × 10 µm × 10 µm cube. Figure 6c represents the superimposition of the radical distributions produced 10^–12^ s after the impact of each particle for doses of 2 Gy deposited by X-rays and by the mixed field obtained in a C-ions SOBP. The OH^●^ production calculated in the cube volume was, respectively, 422,943 radicals in response to 2 Gy X-rays and 519,049 radicals in response to 2 Gy mixed field issued from C-ions SOBP. These data suggest that it is the differences in spatial distribution of ROS rather than the differences in ROS production that explain the differential migration/invasion processes depending on the type of radiation. The profile of OH^●^ distribution in the volume differs strongly between the two types of irradiation: i.e., it is dense and homogenous after X-ray, but it is clustered around the ion trajectories after C-ion irradiation. This profile of ROS spatial distribution may explain the different cell behaviors in response to each type of irradiation; it is as if the ROS concentrated into the C-ion tracks were unable to turn on a switch responsible for activating the signaling pathways and metalloproteases involved in EMT and, consequently, the migration/invasion processes.

## 3. Discussion

The present study first confirmed that HNSCC-CSCs, which are associated with a mesenchymal phenotype, have a higher migration/invasion potential than non-CSCs that display an epithelial phenotype [25]. Their metastatic abilities are increased after X-ray irradiation and decreased with C-ion irradiation under normoxia, as reported previously for several human cancer cell lines [11,26]. Additionally, Matsumoto et al. reported that relative biological effectiveness (RBE) of carbon ions for reducing lung metastasis in a murine model of malignant melanoma are larger than those for cell kill compared to photons [27]. Furthermore, hypoxia significantly enhanced cell migration/invasion, most notably for CSCs, by increasing mesenchymal features (vimentin, N-cadherin), but also the expression of stemness markers (Snail and Twist). These results are consistent with the literature because hypoxic tumor microenvironments favor metastases and CSC maintenance [28]. Under hypoxia, CSCs migrate or invade less in response to X-rays than under normoxia, and C-ion irradiation strengthened this decrease.

The metastatic process requires the degradation of connective tissue associated with vascular basement membranes and interstitial tissue. Matrix metalloproteinases, particularly MMP-2, play a key role in these processes. We observed an increase of MMP-2 activity in CSCs in response to X-rays under normoxia and a decrease under hypoxia, and a decrease in response to C-ions in both oxygen concentrations. These results clearly show that in CSC subpopulations, regulation of migration/invasion in response to radiation is dependent on MMP-2 expression.

Furthermore, HIF-1α, which is expressed under hypoxia and is known to be involved in EMT and in MMP-2 activation, has a role in the response to radiation [9,29]. It has been reported that levels of HIF-1α increase in response to ionizing radiation under both normoxic and hypoxic conditions, whereas no expression was observed after C-ion irradiation under normoxia [9]. In this previous study, we observed the stabilization of HIF-1α with the dose of 10 Gy, which explain that we chose this dose to measure MMP2 activity. We could not latter repeat the experiments at a lower dose due to the lack of beam access. Additionally, the kinetics of ROS production after both irradiation were parallel to the kinetics of HIF-1α expression, suggesting that ROS production and particularly OH^●^ are essential to HIF-1α expression, and particularly in CSCs [9]. Taken together, these data suggest that HIF-1α could partly explain the difference in migration/invasion responses to X-rays and C-ions. It was reported that the upstream activation of HIF-1α is mediated by ROS in response to X-rays or hypoxia [30] and that for C-ion irradiation under normoxia, ROS do not reach the threshold required for HIF-1α stabilization. After HIF-1α silencing, X-ray-induced migration/invasion was abrogated under normoxia and decreased under hypoxia, indicating that HIF-1α is involved in the response to X-rays. Following C-ion irradiation, the decrease in these processes under normoxia was equivalent whether HIF-1α was silenced or not, but it was more important in hypoxia only after silencing of HIF-1α, confirming that the response to C-ions does not involve HIF-1α. The results presented in this study indicate that HIF-1α expression depending on ROS production may explain the differences in invasion/migration between normoxic and hypoxic conditions.

The pathways involved in the invasion/migration abilities under hypoxia and in HNSCC-CSCs have not been elucidated previously. We found that in HNSCC-CSCs under normoxia, the three main signaling pathways (MEK/p38/JNK, Akt/mTOR and STAT3) involved in EMT were activated in response to X-rays. These pathways were associated with the highest migration and invasion abilities and were activated by HIF-1α and MMP-2 expression because after HIF-1α silencing, these processes were inhibited. In response to C-ions, the transduction signals were strongly decreased, irrespective of the dose of irradiation (2 or 10 Gy), and this was associated with poor abilities to migrate and invade. Interestingly, this study also found that when hypoxia was combined with X-ray irradiation, activation of the three pathways was decreased compared with that under normoxia (ratios < 1 for 4 (2 Gy) and 12 (10 Gy) kinases) and the decrease was even greater with C-ion irradiation.

We previously presented evidence that the telomeric status of glioblastoma is important in the response to X-rays, but not to C-ions, a result supported by the differences in the spatial distribution of ROS [31]. In this study, we present additional evidence that a widespread distribution of ROS following X-rays can activate migration/invasion mechanisms. Conversely, ROS that are localized around the ion trajectories after C-ion irradiation should not be able to activate HIF-1α efficiently and, in particular, activate the upstream signaling pathways, which may explain the decrease in the MMP-2 expression and invasion/migration. When ROS production was inhibited with DMSO, the phosphorylation of the studied kinases decreased at both doses of irradiation, a result that confirms that ROS play a key role in the activation of the MAPK, STAT3 and Akt/mTOR pathways. When ROS production is inhibited by DMSO, the migration/invasion induced by X-rays also decreased. Altogether, our data confirm the central role of ROS in the activation of the STAT3, MAPK and Akt/mTOR signaling pathways that initiate the invasion/migration processes through HIF-1α and MMP-2 expression and the importance of their spatial distribution at the nanometric scale (Figure S3).

## 4. Materials and Methods

### 4.1. Cell Culture

SQ20B and FaDu radioresistant cell lines were established from HNSCC tumors and provided by J. Little (Boston, MA, USA) or purchased from the American Type Culture Collection (Manassas, VA, USA). SQ20B and FaDu cell lines, as well as their subpopulations of CSCs, SQ20B-CSCs and FaDu-CSCs, were obtained by flow cytometric cell sorting and cultured as previously described [9,32]. The parental SQ20B cell line, which contains less than 1% CD44-positive cells, was chosen as the non-CSC control cells for SQ20B-CSCs and was named SQ20B^CD44low^. Because the FaDu parental cell line contains more than 20% CD44-positive cells [33], a subpopulation of FaDu CD44-negative cells, named FaDu^CD44low^, was obtained after cell sorting and was used as the non-CSC control cells.

### 4.2. X-ray and C-ion Irradiation

X-ray [32] (250 kV, Lyon-Sud Medical School, Lyon, France) or 290 MeV/n-SOBP C-ion irradiation (LET: 13 keV·µm^–1^) (National Institute of Radiobiological Sciences, Heavy Ions Medical Accelerator of Chiba, Chiba, Japan) were used [34]. Cells were irradiated with both types of irradiation at a dose rate of approximately 2 Gy/min. All the radiobiological parameters calculated according to the linear quadratic model (α, β, RBE at 10% survival) were previously reported for the four subpopulations studied [9,10]. Since RBEs were dependent on the subpopulations tested and conditions of oxygenation, all experiments with C-ions were performed at the equivalent physical dose that was used for X-ray irradiation.

### 4.3. Hypoxic Conditions

Hypoxic conditions were obtained in a tri-gas chamber under an atmosphere containing 1% O_2_, 5% CO_2_ and 94% N_2_ at 37 °C (Heracell 150i; Thermo Fisher Scientific, Waltham, MA, USA, or 9000E; Wakenyaku, Kyoto, Japan). During chronic hypoxia, cells were maintained in hypoxic conditions over several passages before experiments, whereas acute hypoxia corresponded with 0–24 h of hypoxia. During irradiation, flasks with a non-filtered cap were closed and then opened when they were replaced in the incubator. Additionally, during trypsinization, the culture medium was enriched with nitrogen to limit the reoxygenation of the cells.

### 4.4. Transient Transfection

Two siRNAs targeting HIF-1α (exons 5 and 12) and an irrelevant siRNA as a negative control (Thermo Fisher Scientific) were used for transfection experiments. The validation of silencing and the transfection rate were estimated as described by Wozny et al. [9]. The results presented in this paper are only those obtained for the siRNA targeting the exon 5 of HIF-1α.

### 4.5. Quantification of Motility and Cell Migration and Invasion

Cell motility was quantified using a wound-healing assay. The migration/invasion processes were measured using Boyden chambers and xCELLigence for real-time measurement. The IncuCyte wound-healing assay was used for cell migration assays as described by Guy et al. [35]. The data are presented as mean ± SD.

#### 4.5.1. Real-Time Measurement of Cell Migration or Invasion Using xCELLigence

Each well of 16-well plates comprises an upper and a lower chamber separated by a porous membrane containing randomly distributed 8-µm pores and covered with gold microelectrodes at the bottom. Using the RTCA software (version 2.0; Roche Diagnostics, San Diego, CA, USA), the impedance variations were analyzed over 20 h (<cell doubling time) and this was extrapolated to provide a cell index proportional to the migration/invasion process. Before starting the experiments, cells were serum-starved overnight under hypoxic or normoxic conditions with the respective media supplemented with 0.1% bovine serum albumin (BSA). After 16 h, plates were equilibrated for 1 h in the device under normoxic or hypoxic conditions at 37 °C with 165 μL of medium in the lower chamber and 50 μL of serum-free medium 0.1% BSA in the upper chamber. After 1 h, the background signal produced by the cell-free media was measured. A suspension of 30,000 cells in 100 μL of serum-free medium, 0.1% BSA, was seeded in quadruplicate per condition in the upper chambers and remained for 30 min at room temperature to allow the cells to settle onto the membrane. Plates were then placed in the 21% or 1% O_2_ incubator and the cell index was measured every 15 min for 20 h. For the invasion assay, the same protocol was applied, except that Matrigel (BD Biosciences, San Jose, CA, USA; 1/25 in serum-free medium) was layered on the upper side of the membrane. The coated chambers were then incubated for at least 4 h at 37 °C. All subsequent steps were then followed as described for the migration assay. Experiments were repeated three times.

#### 4.5.2. Measurement of Cell Migration or Invasion Using Boyden Chamber Assays

Migration was studied using a 24-well Transwell chamber with randomized 8-μm pore size (Becton Dickinson, Franklin Lakes, NJ, USA). Cells were cultured in 25 cm^2^ flasks for 24 h at a density of 1 × 10^6^ cells per flask under normoxia or hypoxia. Then, cells were starved overnight in medium containing 0.1% BSA instead of fetal bovine serum (FBS). Cells were untreated or treated with a non-cytotoxic concentration of 1% DMSO for 1 h before 0 or 2 Gy X-ray or C-ion irradiation. Cells were then immediately trypsinized and 30,000 cells in 500 μL of serum-free medium 0.1% BSA were transferred to the upper chamber. The lower chamber was filled with medium. After incubation at 37 °C under the normoxic or hypoxic condition (18 h for SQ20B^CD44low^ and SQ20B-CSCs and 15 h for FaDu^CD44low^ and FaDu-CSCs), inserts were fixed and stained using a RAL 555 kit (VWR International, Fontenay-sous-Bois, France). At these times, which were less than the doubling time of each subpopulation (22 h for SQ20B subpopulations and 19 h for FaDu subpopulations), cell cycle analyses did not show a significant percentage of cells in the sub-G1 phase. The number of migrating cells was counted using an inverted optical microscope (×20; Leica Microsystems, Deerfield, IL, USA; Axio Imager; Carl Zeiss Micro Imaging, Jena, Germany). For the invasion assay, Corning BioCoat Growth Factor Reduced BD Matrigel (Becton Dickinson) was used. The protocol used for the migration assay was also applied for the invasion assay except that before adding the cells, coat inserts were equilibrated with 500 µL of no-FBS medium with 0.1% BSA for 6 h at 37 °C. Then, 450 µL of medium were removed from the insert and cells were seeded as described below. Each experiment was performed at least three times in triplicate for each condition.

### 4.6. Western-blot Analyses

After incubation in normoxia or for 6, 12, or 24 h under hypoxia, cells were lysed and the protein concentration was determined (10). Proteins were separated on 10% polyacrylamide gels and transferred onto a nitrocellulose membrane. The monoclonal antibodies used for blotting were: anti-N-cadherin (mouse, 1:3000), anti-E-cadherin (mouse, 1:10,000) and anti-GAPDH (mouse, 1:100,000) (all from BD Transduction, San-Jose, CA, USA), anti-vimentin (mouse, 1:200; Santa Cruz Biotechnology, Santa Cruz, CA, USA), anti-Snail (rabbit, 1:500; Novus Biologicals, Little Town, CO, USA), anti-Twist (mouse, 1:50; Abcam, Cambridge, UK) and HRP-conjugated anti-rabbit and anti-mouse IgG (both 1:7000; Santa Cruz Biotechnology). Western blot signals were measured by densitometric scanning with an Azure C300 Intelligent Dark Box (Biosystems Inc., Dublin, CA, USA). Each Western blot was performed three times.

### 4.7. Quantification of MMP-2 and MMP-9 Expression

Protein samples for determination of MMP-2 and MMP-9 concentrations were obtained as described in the previous section. Quantikine Total MMP-2 and MMP-9 immunoassay kits (R&D Systems, Abingdon, UK) were used to determine MMP concentrations as recommended by the manufacturer, and results were normalized to the total protein concentration in each sample measured using the BCA assay. Each condition was performed at least twice in triplicate.

### 4.8. Determination of the Relative Levels of Protein Phosphorylation

The Human-Phospho-Kinase Array Kit (R&D Systems) was used to compare the phosphorylation profiles of 43 kinases in SQ20B-CSCs and SQ20B^CD44low^ in response to 2 and 10 Gy of both types of irradiation under normoxia and hypoxia. The experimental protocol used was as recommended by the manufacturer. For ROS scavenging, 1% DMSO was added to cells 1 h before irradiation. The chemiluminescence signals were measured by densitometric scanning using an Azure C300 Intelligent Dark Box (Biosystems Inc). The relative protein phosphorylation levels were then quantified using MultiGauge (FujiFilm, Tokyo, Japan) with results expressed as pixels/mm^2^.

### 4.9. ROS Modeling

The primary radicals distribution was obtained by simulating the interactions with liquid water of X-rays or a mixed radiation field issued from a C-ions SOBP. The simulation was composed of three different stages:

The physical stage corresponds to a time period of 10^–15^ s after the impact of each primary particle and leads to a distribution of excited and ionized water molecules and of low energy electrons. This stage is described by the LQD code (LiQuiD water radiolysis) which allows simulation of the interactions of primary particles as well as the electron cascades until thermalization.

The physicochemical stage covers the time period 10^–15^ s to 10^–12^ s and models the relaxation of excited and ionized water molecules as well as the hydration of thermalized electrons. The outcome of the PHYCHEML code is therefore a spatial distribution of the most abundant reactive chemical species (OH^●^, e_aq_, H_3_O^+^, H^●^ and OH^–^).

The last stage is handled by the CHEM code, which models the diffusion and the reactions between these species leading to other chemical species. The details of these three Monte Carlo codes can be found in Gervais et al. [36]_._ Because the energy deposited is highly concentrated along the ion tracks in the case of C-ion irradiation, the distance between radicals is shorter and results in a higher recombination probability. Consequently, the decrease in the total number of hydroxyl radicals is greater for C-ions than for X-rays. For this reason, 10^–12^ s corresponds to the minimum difference between X-rays and C-ions with regards to hydroxyl distribution.

The composition of the mixed radiation field was obtained by simulating the NIRS spread-out Bragg peak in water with the Geant4 software [37]; the fraction of dose deposited by each fragment (specific particle type and energy) in the SOBP position representing the cells’ location was retrieved. This allowed us to consider a particular configuration of the mixed field and perform the three simulation stages presented above for each component.

### 4.10. Statistical Analysis

Variables were compared using the unpaired Student’s *t* test performed with Microsoft Excel (Microsoft, Redmond, WA, USA). Data were considered significant at *p* < 0.05. Levels of significance are indicated by asterisks as follows: * *p* < 0.05; ** *p* < 0.01; *** *p* < 0.001.

## 5. Conclusions

Taken together, our results contribute to the understanding of the underlying mechanisms of the radiation-dependent metastatic abilities of HNSCC-CSCs in a hypoxic tumor microenvironment and how this differs in response to C-ion irradiation compared with X-rays. In addition, this work proposes a new paradigm setting ROS spatial distribution as one of the mechanisms with potentially high relevance to explain the differential responses to X-rays and C-ions for cell invasion and migration. The specific ROS distribution observed in response to C-ions can induce complex DNA lesions and cell death, however it may preserve the plasma membrane and intracellular structures of cells outside the ion tracks and does not allow the achievement of the threshold of ROS that is necessary to activate the signaling pathways involved in migration/invasion.

## Supplementary Materials

The following are available online at https://www.mdpi.com/2072-6694/11/4/468/s1, Figure S1: Impact of acute hypoxia on the EMT phenotypes of CSCs and non-CSCs, Figure S2: Signaling pathways involved in the migration/invasion processes in SQ20B^CD44low^. The phosphorylation levels of the proteins involved in the MEK/p38/JNK, STAT3 and Akt/mTOR pathways were determined in response to 10 Gy X-rays and C-ions ± chronic hypoxia and normalized to the basal conditions for SQ20B-CSCs using the Proteome Profiler Human-Phospho-Kinase Array. A signal > 1 corresponds to an activation of phosphorylation, whereas a signal < 1 is associated with inactivation of the kinases (*n* ≥ 2 in duplicate), Figure S3: Schematic representation of the mechanisms involved in the EMT in response to 10 Gy X-ray and C-ion irradiation under normoxia and chronic hypoxia, Table S1: Phosphorylation signals for each protein involved in the Akt/mTOR, STAT3 and MEK/p38/JNK pathways in SQ20B-CSCs. The values (mean ± SD) were calculated in response to normoxia, chronic hypoxia and 2 Gy X-ray or C-ion irradiation under both normoxia and hypoxia, Table S2: Phosphorylation signals for each protein involved in the Akt/mTOR, STAT3 and MEK/p38/JNK pathways in SQ20B-CSCs. The values (mean ± SD) were calculated in response to normoxia, chronic hypoxia and 10 Gy X-ray or C-ion irradiation under both normoxia and hypoxia, Table S3: Phosphorylation signals for each protein involved in the Akt/mTOR, STAT3 and MEK/p38/JNK pathways in SQ20B^CD44low^. The values (mean ± SD) were calculated in response to normoxia, chronic hypoxia and 10 Gy X-ray or C-ion irradiation under both normoxia and hypoxia, Table S4: Phosphorylation signals for proteins involved in the three signaling pathways in SQ20B-CSCs after DMSO treatment. The values (mean ± SD) were calculated in response to normoxia and 2 Gy X-ray irradiation under normoxia, Table S5: Phosphorylation signals for the proteins involved in the three signaling pathways in SQ20B-CSCs after DMSO treatment. The values (mean ± SD) were calculated in response to normoxia, chronic hypoxia and 10 Gy X-ray or C-ion irradiation under both normoxia and hypoxia, Table S6: Phosphorylation signals for proteins involved in the three signaling pathways in SQ20B^CD44low^ after DMSO treatment. The values (mean ± SD) were calculated in response to normoxia, chronic hypoxia and X-ray and C-ion irradiation under both normoxia and hypoxia.
