# Supplementary material for: ROS Production and Distribution: A New Paradigm to Explain the Differential Effects of X-ray and Carbon Ion Irradiation on Cancer Stem Cell Migration and Invasion
Source: Cancers (Basel). 2019 Apr 3;11(4):468. doi: 10.3390/cancers11040468 (PMC6521340; doi:10.3390/cancers11040468)
Supplement: Supplementary file 1 [file cancers-11-00468-s001.pdf]

cancers

MDPI

Supplementary Materials: ROS Production and Distribution: A New Paradigm to Explain the Differential Effects of X-ray and Carbon Ion Irradiation on Cancer Stem Cell Migration and Invasion

Anne-Sophie Wozny, Guillaume Vares, Gersende Alphonse, Alexandra Lauret, Caterina Monini, Nicolas Magné, Charlotte Cuerq, Akira Fujimori, Jean-Claude Monboisse, Michael Beuve, Tetsuo Nakajima and Claire Rodriguez-Lafrasse

Table S1. Phosphorylation signals for each protein involved in the Akt/mTOR, STAT3, and MEK/p38/JNK pathways in SQ20B-CSCs. The values (mean ± SD) were calculated in response to normoxia, chronic hypoxia, and 2 Gy X-ray or C-ion irradiation under both normoxia and hypoxia.

	**Normoxia**		**Normoxia + X-rays**		**Normoxia + C-ions**		**Hypoxia**		**Hypoxia + X-rays**		**Hypoxia + C-ions**	
	Mean	SD	Mean	SD	Mean	SD	Mean	SD	Mean	SD	Mean	SD
**p38a**	13.615	0.191	21.323	0.342	10.057	0.410	14.358	0.485	18.452	0.699	14.134	0.279
**ERK1/2**	12.805	0.233	15.001	1.214	9.157	0.319	12.771	0.532	14.254	1.228	12.792	0.162
**JNK1/2/3**	15.550	0.170	20.474	0.394	17.465	0.621	21.719	0.337	18.579	0.023	18.132	0.404
**MSK1/2**	15.365	0.276	19.259	2.640	11.391	0.379	15.850	0.378	18.958	3.233	15.943	0.985
**c-jun**	13.805	0.049	15.993	0.006	13.592	0.334	14.069	0.153	13.972	0.511	14.654	0.369
**WNK-1**	35.580	2.206	31.568	1.061	34.842	0.539	30.522	0.343	34.541	1.574	31.935	2.472
**GSK-3α/β**	17.780	0.905	19.483	0.439	16.881	1.170	20.033	0.413	20.298	2.129	16.307	0.603
**HSP27**	12.810	0.750	11.326	0.187	8.334	0.205	11.729	0.464	12.951	0.023	12.704	0.478
**STAT3 Y705**	14.455	0.276	14.886	0.056	12.784	0.035	14.625	0.106	13.885	0.802	16.136	0.101
**STAT3 S727**	18.230	1.365	23.460	0.534	15.338	0.813	16.908	0.337	21.777	1.336	17.718	0.196
**β-catenin**	14.035	0.064	14.444	0.820	9.434	0.265	13.866	0.907	13.451	0.528	13.416	1.426
**Akt1/2/3 S473**	13.290	0.757	14.535	0.646	8.850	0.308	13.177	0.140	13.298	0.001	12.709	0.412
**Akt1/2/3 T308**	20.035	1.379	14.294	0.075	12.909	0.000	15.892	0.118	13.830	0.196	18.303	2.147
**mTOR**	15.110	0.778	15.749	0.439	12.407	1.345	14.507	0.091	15.331	0.381	14.706	0.029
**PRAS40**	32.020	1.315	32.046	2.711	31.911	3.112	31.888	1.546	51.658	2.199	27.212	1.096
**p70 S6 kinase**	16.285	0.445	13.707	0.223	11.667	0.217	14.028	0.112	11.611	0.277	14.998	0.291

Table S2. Phosphorylation signals for each protein involved in the Akt/mTOR, STAT3, and MEK/p38/JNK pathways in SQ20B-CSCs. The values (mean ± SD) were calculated in response to normoxia, chronic hypoxia, and 10 Gy X-ray or C-ion irradiation under both normoxia and hypoxia.

	**Normoxia**		**Normoxia + X-rays**		**Normoxia + C-ions**		**Hypoxia**		**Hypoxia + X-rays**		**Hypoxia + C-ions**	
	Mean	SD	Mean	SD	Mean	SD	Mean	SD	Mean	SD	Mean	SD
**p38a**	4.275	0.212	8.060	1.050	0.980	0.170	2.249	1.159	3.120	0.815	0.425	0.085
**ERK1/2**	2.320	0.297	3.753	0.000	0.350	0.057	2.250	0.028	1.204	0.175	0.130	0.010
**JNK1/2/3**	4.550	0.099	4.655	0.229	1.005	0.629	4.260	0.099	4.458	1.060	1.325	0.005
**MSK1/2**	12.625	1.803	16.234	2.396	7.620	1.725	14.262	0.318	10.718	0.630	6.035	0.765
**c-jun**	1.805	0.219	2.774	0.010	0.795	0.049	1.696	0.410	1.724	0.030	0.730	0.040
**WNK-1**	19.210	0.679	16.308	1.069	2.815	0.092	17.170	0.148	23.918	0.685	1.660	0.040
**GSK-3α/β**	6.325	0.163	7.094	0.220	1.195	0.502	6.664	0.240	7.157	1.050	1.575	0.095
**HSP27**	2.225	0.049	2.653	0.162	0.565	0.276	1.565	0.198	1.075	0.010	0.485	0.075
**STAT3 Y705**	1.595	0.290	1.613	0.200	0.385	0.120	0.969	0.099	1.131	0.130	0.175	0.125
**STAT3 S727**	1.675	0.629	1.964	0.449	1.445	0.021	1.350	0.049	1.383	0.375	1.495	0.475
**β-catenin**	12.855	0.120	10.348	0.506	1.545	0.092	15.303	0.049	16.929	1.105	2.645	0.085
**Akt1/2/3 S473**	2.265	0.544	3.301	0.353	0.185	0.021	2.385	0.014	2.436	0.715	0.315	0.055
**Akt1/2/3 T308**	3.000	0.495	3.524	0.477	0.740	0.014	3.011	0.304	3.080	0.650	0.680	0.150
**mTOR**	1.365	0.064	3.112	0.048	0.235	0.205	0.571	0.035	0.258	0.010	0.335	0.135
**PRAS40**	40.055	1.421	71.408	7.990	24.125	0.601	54.585	2.560	57.148	1.655	23.230	3.200
**p70 S6 kinase**	2.645	0.120	2.592	0.286	1.920	0.269	1.297	0.346	1.826	0.310	2.965	0.495

Table S3. Phosphorylation signals for each protein involved in the Akt/mTOR, STAT3, and MEK/p38/JNK pathways in SQ20B^{CD44low}. The values (mean ± SD) were calculated in response to normoxia, chronic hypoxia, and 10 Gy X-ray or C-ion irradiation under both normoxia and hypoxia.

	**Normoxia**		**Normoxia + X-rays**		**Normoxia + C-ions**		**Hypoxia**		**Hypoxia + X-rays**		**Hypoxia + C-ions**	
	Mean	SD	Mean	SD	Mean	SD	Mean	SD	Mean	SD	Mean	SD
**p38a**	2.019	0.374	2.058	0.068	1.341	0.442	1.207	0.211	0.784	0.184	1.389	0.646
**ERK1/2**	1.327	0.041	1.442	0.136	1.115	0.014	0.942	0.095	0.788	0.095	0.736	0.034
**JNK1/2/3**	1.476	0.061	1.740	0.041	0.851	0.007	1.442	0.177	1.375	0.000	1.481	0.136
**MSK1/2**	5.591	0.360	5.966	0.388	3.005	0.238	5.279	0.367	4.957	0.483	4.639	0.075
**c-jun**	1.346	0.082	1.010	0.014	1.462	0.068	0.865	0.109	0.692	0.000	0.462	0.000
**WNK-1**	5.548	0.163	5.495	0.156	2.582	0.129	4.149	0.129	4.188	0.197	3.957	0.197
**GSK-3α/β**	2.399	0.061	2.404	0.394	1.577	0.095	2.197	0.170	1.880	0.374	1.990	0.462
**HSP27**	0.889	0.143	0.933	0.054	0.413	0.190	1.139	0.116	0.909	0.034	0.543	0.184
**STAT3 Y705**	0.957	0.170	0.380	0.075	1.144	0.320	0.582	0.184	0.620	0.197	0.389	0.197
**STAT3 S727**	0.976	0.238	0.457	0.197	1.259	0.136	0.404	0.041	0.736	0.007	0.505	0.007
**β-catenin**	7.490	0.231	7.702	0.258	2.635	0.218	7.779	0.136	7.481	0.163	3.918	0.020
**Akt1/2/3 S473**	0.788	0.054	0.942	0.027	0.596	0.068	0.875	0.014	0.740	0.054	0.870	0.279
**Akt1/2/3 T308**	1.635	0.503	1.168	0.197	0.678	0.197	1.043	0.088	1.192	0.027	0.962	0.027
**mTOR**	0.500	0.000	0.784	0.034	0.538	0.238	0.649	0.061	0.428	0.048	0.476	0.088
**PRAS40**	8.788	0.639	8.702	0.490	6.231	0.367	8.697	0.469	7.365	1.319	7.135	1.319
**p70 S6 kinase**	1.135	0.190	0.630	0.020	1.361	0.592	0.457	0.007	0.688	0.007	0.457	0.007

Table S4. Phosphorylation signals for proteins involved in the three signaling pathways in SQ20B-CSCs after DMSO treatment. The values (mean ± SD) were calculated in response to normoxia and 2 Gy X-ray irradiation under normoxia.

	**Normoxia**		**Normoxia + X-rays**	
	Mean	SD	Mean	SD
**p38a**	7.530	1.159	2.775	0.158
**ERK1/2**	3.945	0.445	2.158	0.153
**JNK1/2/3**	8.235	0.120	6.317	0.439
**MSK1/2**	10.265	1.846	5.202	0.270
**c-jun**	1.650	0.325	1.732	0.143
**WNK-1**	3.950	0.353	3.766	0.471
**GSK-3α/β**	6.03	0.141	4.495	0.085
**HSP27**	2.77	0.085	1.627	0.069
**STAT3 Y705**	1.565	0.163	1.245	0.418
**STAT3 S727**	4.555	0.007	2.44222	0.037
**β-catenin**	4.165	0.092	2.936	0.100
**Akt1/2/3 S473**	7.570	0.198	3.961	0.354
**Akt1/2/3 T308**	1.590	0.014	2.128	0.164
**mTOR**	4.170	0.254	2.809	0.291
**PRAS40**	12.820	1.570	9.922	0.048
**p70 S6 kinase**	1.185	0.0494	1.174	0.159

Table S5. Phosphorylation signals for proteins involved in the three signaling pathways in SQ20B-CSCs after DMSO treatment. The values (mean ± SD) were calculated in response to normoxia, chronic hypoxia, and 10 Gy X-ray or C-ion irradiation under both normoxia and hypoxia.

	**Normoxia**		**Normoxia + X-rays**		**Normoxia + C-ions**		**Hypoxia**		**Hypoxia + X-rays**		**Hypoxia + C-ions**	
	Mean	SD	Mean	SD	Mean	SD	Mean	SD	Mean	SD	Mean	SD
**p38a**	4.755	0.092	3.035	0.120	5.035	0.035	3.395	0.106	4.230	0.509	3.475	0.085
**ERK1/2**	4.205	0.163	2.965	0.134	3.590	0.000	3.555	0.120	3.545	0.021	3.625	0.078
**JNK1/2/3**	7.945	0.205	8.220	0.014	8.065	0.092	8.325	0.120	8.435	0.573	9.110	0.057
**MSK1/2**	12.320	0.467	11.355	0.841	11.120	0.834	11.940	0.141	12.860	0.820	10.535	2.029
**c-jun**	6.590	0.283	3.645	0.007	4.560	0.085	2.350	0.014	2.650	0.000	3.590	0.057
**WNK-1**	24.755	0.940	22.530	0.198	20.315	0.742	15.965	0.148	27.995	0.672	13.680	0.042
**GSK-3α/β**	12.340	0.424	8.120	0.042	7.680	0.141	13.480	0.636	10.120	0.085	9.230	0.212
**HSP27**	4.705	0.247	4.350	0.028	4.075	0.106	4.265	0.389	4.265	0.389	4.470	0.113
**STAT3 Y705**	3.625	0.134	2.495	0.064	3.160	0.198	3.635	0.389	2.760	0.226	2.730	0.156
**STAT3 S727**	4.410	0.933	3.055	0.474	2.895	0.290	3.330	0.198	3.055	0.431	2.915	0.276
**β-catenin**	20.510	0.099	10.815	0.049	6.685	0.191	21.435	0.318	15.030	0.622	5.925	0.049
**Akt1/2/3 S473**	7.900	0.523	7.500	0.014	5.775	0.106	7.605	0.403	11.200	0.184	6.720	0.170
**Akt1/2/3 T308**	5.835	1.393	2.945	0.615	3.705	0.516	5.775	2.482	3.485	0.601	3.815	0.870
**mTOR**	6.390	0.834	5.240	0.240	5.625	0.134	7.630	0.057	5.755	0.177	6.975	0.064
**PRAS40**	42.730	0.792	41.505	1.322	33.440	1.287	47.260	1.640	32.585	0.460	35.155	2.072
**p70 S6 kinase**	5.050	0.099	2.545	0.049	3.285	0.191	4.065	0.219	3.410	0.268	3.130	0.028

Table S6. Phosphorylation signals for proteins involved in the three signaling pathways in SQ20B^{CD44low} after DMSO treatment. The values (mean ± SD) were calculated in response to normoxia, chronic hypoxia, and X-ray and C-ion irradiation under both normoxia and hypoxia.

	**Normoxia**		**Normoxia + X-rays**		**Normoxia + C-ions**		**Hypoxia**		**Hypoxia + X-rays**		**Hypoxia + C-ions**	
	Mean	SD	Mean	SD	Mean	SD	Mean	SD	Mean	SD	Mean	SD
**p38a**	2.181	0.011	1.237	0.451	0.500	0.087	1.118	0.033	1.530	0.633	0.231	0.065
**ERK1/2**	1.184	0.151	1.048	0.155	0.179	0.029	1.121	0.013	0.590	0.136	0.071	0.007
**JNK1/2/3**	2.321	0.051	1.882	0.296	0.513	0.321	2.118	0.259	2.187	0.824	0.720	0.004
**MSK1/2**	6.441	0.920	5.239	0.087	3.888	0.880	7.092	0.665	5.258	0.490	3.279	0.588
**c-jun**	0.921	0.111	0.599	0.054	0.406	0.025	0.843	0.213	0.846	0.506	0.397	0.031
**WNK-1**	9.801	0.346	7.625	0.703	1.436	0.047	8.541	0.825	11.734	0.533	0.902	0.031
**GSK-3α/β**	3.227	0.083	2.987	0.378	0.610	0.256	3.313	0.184	3.511	0.815	0.556	0.073
**HSP27**	1.135	0.025	0.770	0.022	0.288	0.141	0.778	0.079	0.528	0.008	0.266	0.058
**STAT3 Y705**	0.813	0.148	0.862	0.621	0.196	0.061	0.482	0.289	0.555	0.101	0.095	0.096
**STAT3 S727**	0.854	0.322	0.474	0.072	0.737	0.011	0.672	0.263	0.679	0.291	0.821	0.365
**β-catenin**	6.558	0.061	5.221	0.415	0.788	0.047	7.609	0.151	8.304	0.859	1.438	0.651
**Akt1/2/3 S473**	1.156	0.278	1.080	0.223	0.087	0.011	1.071	0.163	0.803	0.556	0.141	0.042
**Akt1/2/3 T308**	1.536	0.252	1.268	0.292	0.378	0.007	1.497	0.326	1.511	0.505	0.370	0.115
**mTOR**	0.696	0.032	1.275	0.122	0.120	0.105	0.284	0.008	0.126	0.008	0.182	0.104
**PRAS40**	28.605	1.122	24.834	0.032	12.309	0.307	27.140	1.515	28.036	1.286	12.625	2.460
**p70 S6 kinase**	1.349	0.061	0.956	0.063	0.979	0.137	0.645	0.184	0.896	0.241	1.611	0.380

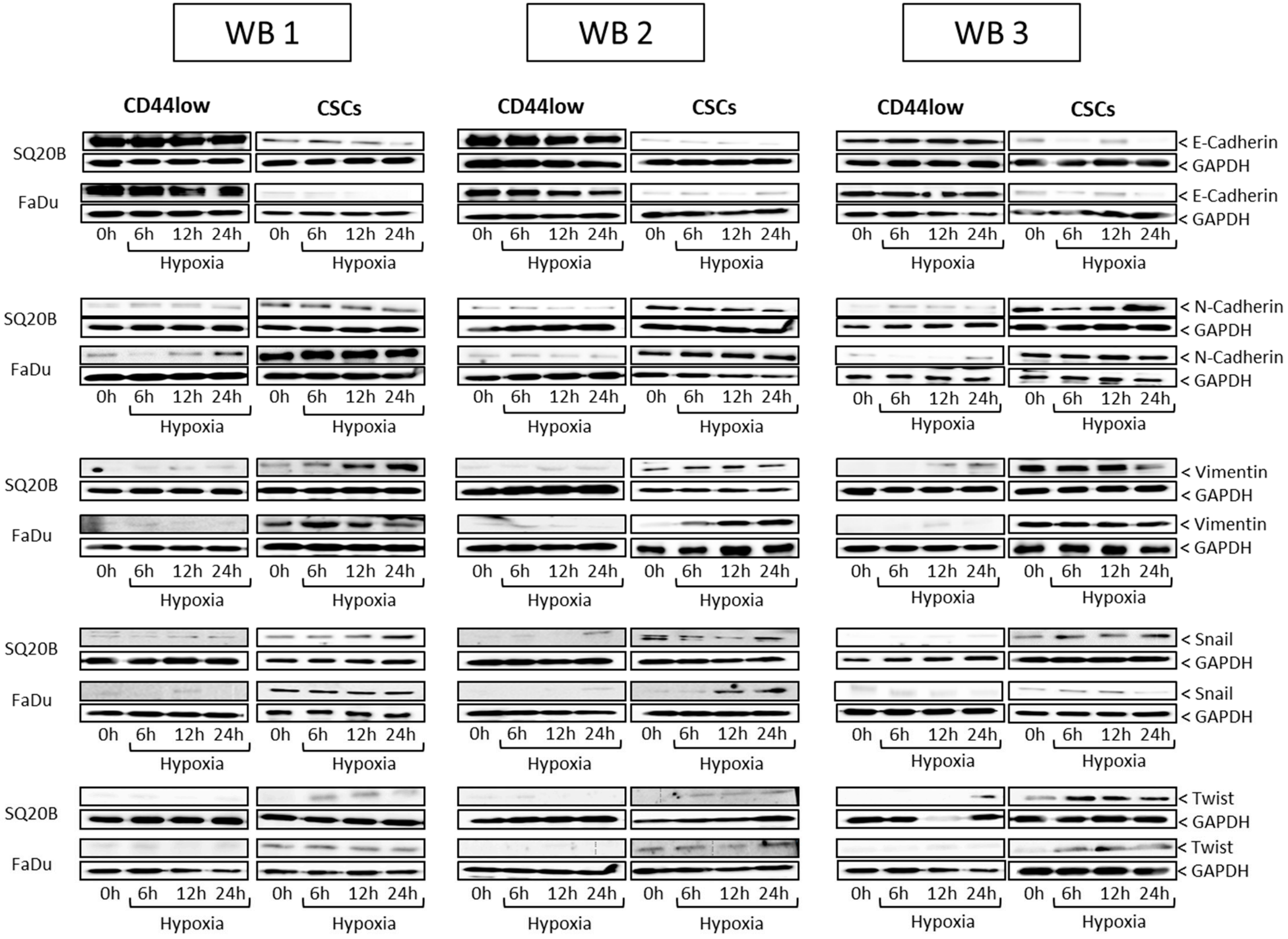

Figure S1. Impact of acute hypoxia on the EMT phenotypes of CSCs and non-CSCs.

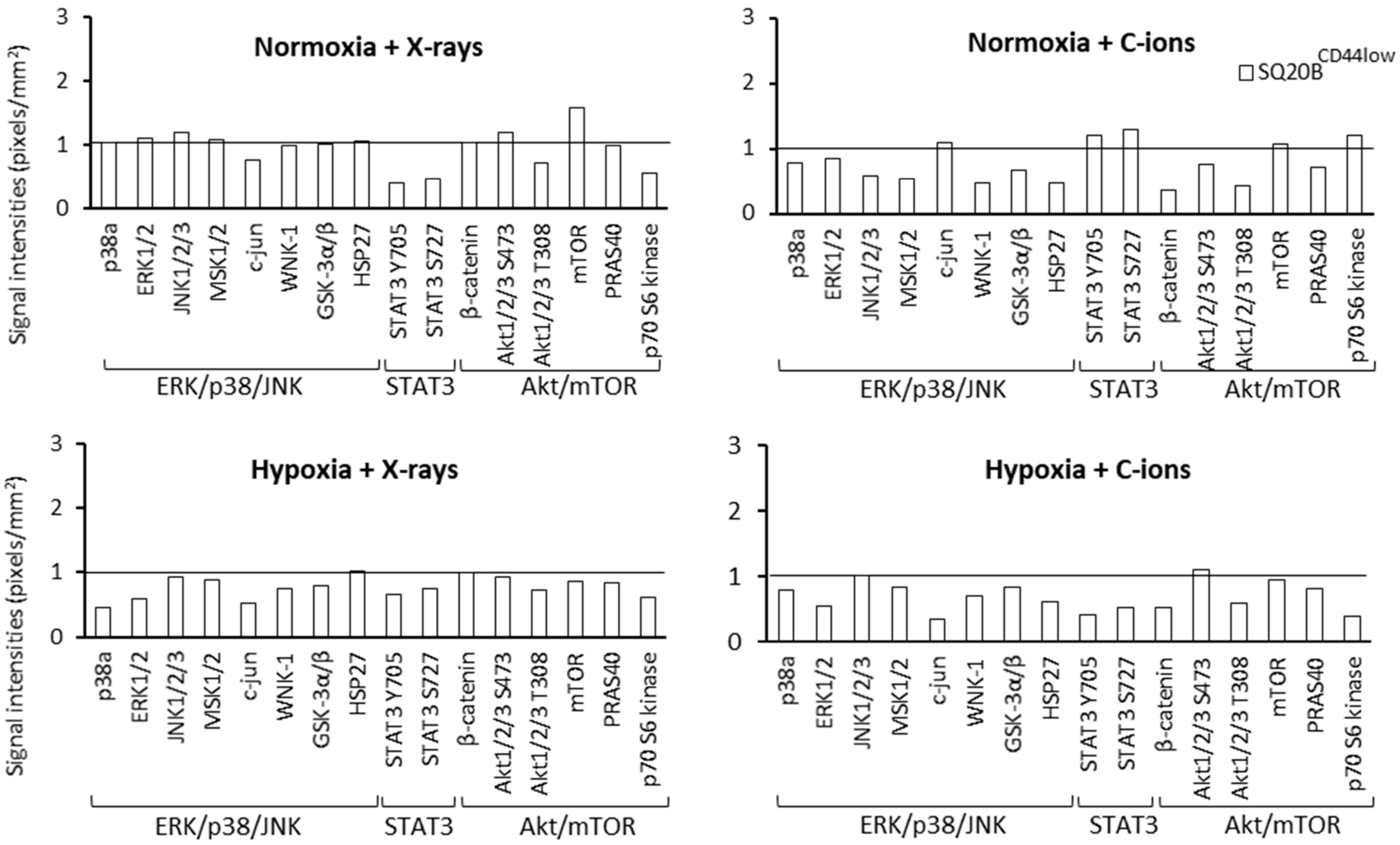

Figure S2. Signaling pathways involved in the migration/invasion processes in SQ20B[CD44Low]. The phosphorylation levels of the proteins involved in the MEK/p38/JNK, STAT3, and Akt/mTOR pathways were determined in response to 10 Gy X-rays and C-ions ± chronic hypoxia and normalized to the basal conditions for SQ20B-CSCs using the Proteome Profiler Human-Phospho-Kinase Array. A signal > 1 corresponds to an activation of phosphorylation whereas a signal < 1 is associated with inactivation of the kinases ($n \geq 2$ in duplicate).

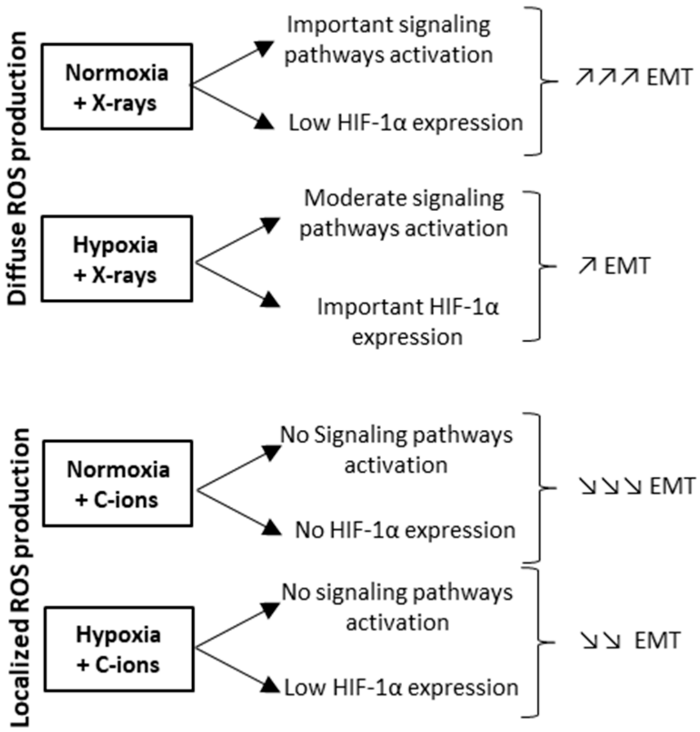

Figure S3. Schematic representation of the mechanisms involved in the EMT in response to 10 Gy X-ray and C-ion irradiation under normoxia and chronic hypoxia.

© 2019 by the authors. Licensee MDPI, Basel, Switzerland. This article is an open access article distributed under the terms and conditions of the Creative Commons Attribution (CC BY) license (http://creativecommons.org/licenses/by/4.0/).
